# A laboratory driving simulation for assessment of driving behavior in adults with ADHD: a controlled study

**DOI:** 10.1186/1744-859X-6-4

**Published:** 2007-01-30

**Authors:** Joseph Biederman, Ronna Fried, Michael C Monuteaux, Bryan Reimer, Joseph F Coughlin, Craig B Surman, Megan Aleardi, Meghan Dougherty, Steven Schoenfeld, Thomas J Spencer, Stephen V Faraone

**Affiliations:** 1 Pediatric Psychopharmacology Department at Massachusetts General Hospital in Boston, MA, USA; 2 Department of Psychiatry at the Harvard Medical School, Boston MA, USA; 3 MIT Center for Transportation & Logistics, 77 Massachusetts Avenue, E40-276 Cambridge, MA 02139, USA; 4 Medical Genetics Research Center and Department of Psychiatry, SUNY Upstate Medical University, Syracuse, NY, USA

## Abstract

**Background:**

It is now estimated that attention deficit-hyperactivity disorder (ADHD) afflicts at least 4% of adults in the United States and is associated with high levels of morbidity and functional impairment. One key area of dysfunction associated with ADHD is impaired motor vehicle operation. Our goal was to examine the association between ADHD and specific driving outcomes in a sample of adults using a driving simulator.

**Methods:**

Subjects were 20 adults with full DSM-IV ADHD and 21 controls without ADHD of equal gender distribution. However, the mean age of subjects with ADHD was somewhat older. All analyses were adjusted for age and gender. All subjects participated in a driving simulation that lasted for one hour and consisted of a short training period, a high stimulus segment and a low stimulus segment with two distinct monotonous periods.

**Results:**

In the second monotonous period within the low stimulus environment, ADHD subjects were significantly more likely than controls to collide with an obstacle suddenly appearing from the periphery, adjusting for age and gender.

**Conclusion:**

Adults with ADHD were more likely than controls to collide with an obstacle during a driving simulation suggesting that deficits in directed attention may underlie driving impairments in this population.

## Background

It is now estimated that Attention Deficit Hyperactivity Disorder (ADHD) persists in a substantial number of cases [1] and afflicts at least 4% of adults in the United States [2,3]. Emerging data from clinical and community samples document that ADHD in adults is associated with high levels of morbidity and functional impairment [4,5]. One key area of dysfunction associated with ADHD is impaired motor vehicle operation. Driving accidents are a major public health concern being one of the leading causes of death in the United States as of 2003 [6].

An emerging literature shows that drivers with ADHD are more likely than drivers without ADHD to commit traffic violations and have adverse driving outcomes. A follow-up of adolescents and young adults with and without ADHD found those with ADHD were more likely to be involved in traffic accidents, to be at fault in automobile accidents and to have received more traffic citations than controls [7]. Another follow-up study reported that ADHD subjects were more likely to incur more significant damage to their vehicles in crashes than normal controls [8] indicating more severe accidents. Likewise, two epidemiological longitudinal studies of New Zealander adolescents reported that individuals with ADHD had significantly more driving transgressions than controls [9] and were at a greater risk to have traffic accidents involving injuries and traffic violations [10].

Furthermore, documentation through surveys and RMV questionnaires has shown that individuals with ADHD have worse driving histories than subjects with other psychiatric disorders [11] and display significantly worse safe driving habits than matched controls [7,12]. Reimer et al. [13] compared the driving behavior of subjects with and without ADHD on the Manchester Driving Behavior Questionnaire (DBQ) and a driving history questionnaire. Results documented that adult ADHD subjects scored significantly worse than non-ADHD subjects on all aspects indexing impaired driving behavior. Although very informative, these questionnaire-based studies provide limited understanding as to the underlying components of impaired driving associated with ADHD.

In recent years driving simulator paradigms have been developed to help quantify key components of driving behaviors among individuals with ADHD [14,15]. These studies failed to fully establish the validity of the driving simulation paradigm used, [16] making it difficult to interpret their results.

To address the limitations of the extant literature, our group has developed and validated a novel driving simulator paradigm for the study of impaired driving behavior in individuals with ADHD [17]. Convergent validity was assessed through a comparison of self-reported behaviors such as traffic tickets and accidents with simulated outcomes; discriminate validity was assessed through a multi-trait, multi-method matrix and concurrent validity was established through the analysis of accident rates between ADHD subjects and controls in the driving simulator.

This new paradigm was designed to assess the core components of the clinical picture of ADHD that could account for driving impairments in individuals with ADHD. Therefore proxies of impulsivity, hyperactivity and inattention were incorporated into the development of the driving paradigm. To assess inattention, the driving simulation included a monotonous driving period punctuated with a sudden peripheral stimulus (a dog running into the road). Such a setting was designed to mimic a non-stimulating section of highway situation whereby individuals with ADHD become bored and become less attentive. To assess impulsivity, the simulator included a slow moving lead vehicle and assessed whether the drivers could tolerate not passing the vehicle through the frequency of lane departures indexing passing attempts. Hyperactivity was assessed through the logging of driving speed throughout the simulation as well as rate of stopping before a traffic light or stop sign.

The main purpose of this study was to evaluate the driving impairments in individuals with ADHD using this new well-developed and ecologically valid paradigm. We hypothesized that ADHD drivers will show specific driving impairments associated with the proxies indexing the core features of ADHD: inattention, hyperactivity and impulsivity.

## Methods

### Subjects

Subjects were 20 adults with DSM-IV ADHD and 21 controls without ADHD of equal gender distribution. However, the mean age of subjects with ADHD was somewhat older. All analyses were adjusted for age and gender similar age and sex. All ADHD subjects met full DSM-IV criteria and had symptom onset in childhood and persistent symptomatology into adulthood. ADHD subjects with and without prior histories of treatment for the condition were included. Controls were included if they failed to meet criteria for ADHD and endorsed fewer than 3 ADHD symptoms at any level of severity. Participants were required to be English speakers. Excluded were subjects with an IQ less than 80. Subjects were recruited through clinical referrals to an adult ADHD program at a major medical center and through advertisement in local media. The local institutional review board approved this study and all subjects provided written informed consent for participation.

### Clinical assessments

Diagnostic assessment relied on the Structured Clinical Interview for DSM-IV [18] supplemented for childhood disorders by modules from the Kiddie Schedule for Affective Disorders and Schizophrenia for School-Age Children-Epidemiologic Version [19]. Raters performing assessments and interviews were blind to the ascertainment status of the probands. Socioeconomic status (SES) was assessed with the Hollingshead four-factor scale [20].

To have been given a full diagnosis of adult ADHD, the subject must have (1) met full DSM-IV criteria (at least 6 of 9 symptoms) for inattentive or hyperactive/impulsive subtypes, with the onset of multiple symptoms by age 7 years; (2) described a chronic course of ADHD symptoms from childhood to adulthood; and (3) endorsed a moderate or severe level of impairment attributed to the ADHD symptoms.

To elicit ADHD symptomatology, we used the ADHD module from the Kiddie SADS-E, wording questions to inquire if symptoms in childhood were currently present, and then asking whether the symptoms were present currently to a clinically meaningful degree. By using this method, we assured that the syndrome observed in adulthood had some continuity with the syndrome reported in childhood.

### Driving simulator

The driving simulator used an instrumented full cab 2001 Volkswagen Beetle with a front projection screen that provided the driver with a near 40-degree view of virtual roadway. The original equipment manufacture (OEM) accelerator, brake, and steering wheel recorded inputs from the driver. Driving simulation software used STISEM Drive and STISIM Open Module to compute graphical updates, based upon a validated driving simulation [17]. The driving simulation lasted for one hour and consisted of three segments consisting of a training period, a high-stimulus testing period and a low-stimulus testing period. There was a slight pause between each segment.

The training segment provides subjects with an approximately 10 minute driving period to become acclimated to the simulated driving environment [17]. The protocol was designed so that a driver closely monitoring the appropriate speed limit would drive for approximately 45 minutes. The high stimulus portion of the protocol included features of urban driving, such as traffic lights, stop signs, jaywalkers and parked cars pulling out into traffic. Posted speed limits were between 45 and 35 MPH. The car-following condition introduced low-stimulus driving with oncoming traffic to constrain the driver to follow a lead vehicle with a velocity changing according to a sinusoidal function with mean 35 mph. The behaviors measured during this period were designed as a proxy of impulsivity. In the low stimulus portion of the protocol two "monotonous" segments, described in more detail below, are separated by a rural road with hills and curves and a four lane highway. The rural road included incidences where an on coming vehicle suddenly invaded the driver's lane and a slow vehicle appeared in front of the driver. The highway provided drivers with the opportunity to follow or pass surrounding traffic. The posted speed limits in the rural and highway sections were 55 MPH and 65 MPH respectively. For further details on the components of the simulation refer to Reimer et al. [17].

As mentioned earlier, the first and last portions of the low-stimulus testing period included "monotonous" segments where the driver travels for two miles along a two-lane highway with a posted speed limit of 55 MPH without oncoming traffic. The low-stimulus segments of monotonous driving were punctuated with a sudden peripheral stimulus in the form of a dog, which appears 60 feet off the right side of the roadway and 350 feet ahead. The dog moved toward the road at 18 feet per second. To avoid impacting driver's subsequent performance by a collision, no major action was required to avoid collision in the first presentation of the dogs. During the second presentation of the stimulus, however, the timing of the dogs was such that major evasive action was required to avert a collision.

When entering the car, drivers were instructed on procedures for adjusting the seat, the visual presentation of the rearview mirror and using the OEM speedometer. Participants were told that they would receive a base compensation of $40 for their participation. Finally, to encourage subjects to obey traffic laws, while balancing the need to drive at an appropriate speed, a two part financial incentive structure was presented [17,21]. Participants were instructed that they had 45 minutes to complete the experimental portion of the simulation and that $1 would be deducted from a $20 bonus for each minute they were late. Additionally, participants were told that they would be penalized $5 for each crash and $1 for each traffic ticket (speeding, reckless driving, running stop signs and traffic lights) from a second $20 bonus.

Driver performance was categorized across a number of parameters: velocity, lane position, reaction time and a binary measure of collision with the sudden peripheral stimulus. Velocity was measured as the maximum velocity reached by the driver, relative to the posted speed limit. This aspect of driving behavior was measured as a proxy of hyperactivity. Lane position was measured as the variability of the driver's position within lane. Reaction time was measured as the time from the start of any sudden movement of the steering wheel or depression of the brake pedal and rate of stopping before a traffic light or stop sign was also designed to be a proxy of hyperactivity.

### Statistical analyses

Demographic variables were analyzed using two-sample t-tests and Pearson's chi-squared test for continuous and binary outcomes, respectively. Driving outcomes were assessed with a series of linear and logistic regression models for continuous and binary outcomes, respectively, with the driving outcome as the dependent variable and ADHD status and any confounding factors as the independent variables. As an exploratory analysis, we repeated these regressions, modeling the driving outcomes as a function of ADHD status, gender, and the ADHD-by-gender interaction. The interaction tests if the association between ADHD and the driving outcome differs by gender. If the interaction term is significant, we estimated the ADHD effect within stratum of gender. This interaction analysis was repeated using ADHD status, age (18-2 versus 27–51) and the ADHD-by-age interaction as independent variables. Alpha was set at 0.01 to avoid type I errors and all tests were two-tailed.

## Results

### Demographic Characteristics

The gender distribution did not differ between ADHD and control subjects (percent male: 50% versus 52%, respectively; χ^2^_(1) _< 0.0, p = 0.88). However, the mean age of ADHD subjects was somewhat older than controls (32.0 ± 8.0 versus 27.2 ± 7.5; t(39) = -2.0, p = 0.06).

### Driving Outcomes

Comparisons between ADHD and Control subjects are displayed in table 1. As shown, there were no differences between ADHD and control subjects on measures of velocity, lane position, reaction time or collisions during the initial monotonous period. However, in the second monotonous period ADHD subjects were significantly more likely than controls to collide with an obstacle, suddenly appearing from the periphery, adjusting for age and gender.

**Table 1:** Driving outcomes in ADHD and control adults in low stimulus segment of simulated driving assessment

Driving Outcome	Controls n = 21	ADHD n = 20	Test Statistic (df), p value
Initial Monotonous Period			

Maximum Velocity	59.6 ± 4.1	59.8 ± 2.6	t(40) = -0.2, p = 0.85
Mean Velocity	55.9 ± 3.8	56.0 ± 2.3	t(40) < 0.1, p = 0.99
Lane Position	0.62 ± 0.2	0.65 ± 0.2	t(40) = 0.5, p = 0.61
Reaction Time	1.99 ± 0.6	2.05 ± 0.4	t(40) = 0.1, p = 0.93

Final Monotonous Period			

Maximum Velocity	58.4 ± 6.0	58.4 ± 4.0	t(40) = -0.3, p = 0.81
Mean Velocity	55.3 ± 5.5	54.9 ± 3.2	t(40) = -0.6, p = 0.58
Lane Position	0.65 ± 0.1	0.65 ± 0.2	t(40) = -0.4, p = 0.69
Reaction Time	1.73 ± 0.8	2.10 ± 0.4	t(36) = 1.4, p = 0.18
Collision	6(29)	13(65)	χ^2^_(1) _= 4.0, p = 0.05

df = degrees of freedom

Values in table represent *mean *± *standard deviation *or *frequency (percent)*. All analyses adjusted for age and gender

As an exploratory analysis, we also estimated the driving outcomes as a function of ADHD, gender, and the ADHD status-by-gender interaction. None of the interaction terms were significant, providing no evidence that the association between ADHD and driving performance differed by the gender of the subject. Finally, we estimated the driving outcomes as a function of ADHD, age, and the ADHD status-by-gender interaction. Again, none of the interaction terms were significant, providing no evidence that the association between ADHD and driving performance differed by the age of the subject.

## Discussion

The main purpose of this study was to evaluate the informativeness of a new validated driving paradigm in understanding driving impairments in individuals with ADHD. Our results indicated that ADHD subjects were selectively more impaired than controls in the component of the driving paradigm designed to assess impairments in attention. These results support the hypothesis that inattention is a key moderator of impaired driving behavior in ADHD.

The strengths of this report include the well-characterized sample, blindness and the reliance on a sophisticated, ecologically valid simulator. This is especially important since very few other studies have been able to document consistent results from a driving simulation.

Our results showing that individuals with ADHD had evidence of inattention that occurred selectively during a low stimulus environment punctuated by a sudden unexpected stimulus are consistent with what has been hypothesized to be a key factor in poor driving performance in individuals with ADHD, but not tested in a simulator. The impairment observed is consistent with our hypothesis that individuals with ADHD have difficulties to remain alert while driving without stimulation. Such results are consistent with current neuropsychological conceptualization of ADHD as a disorder of directed attention and not generalized nonspecific inattention. Thus, having the simulation designed to involve a period of monotonous driving with an unanticipated event confirms the study hypothesis and supports the heuristic value of the paradigm used.

Anticipating and being alert to the unexpected has also been documented as a deficit in individuals with ADHD through Continuous Performance Tests (CPT) [22,23]. Moreover, these results are also consistent with a body of literature documenting that individuals with ADHD have impairments on the CPT. Like our driving paradigm, this test is also designed to create a monotonous situation whereby individuals with ADHD become bored and become less attentive [12] and less alert to the unexpected [22,23]. Although there are a number of measures within CPT tasks that point to inattention as being a primary factor in the impaired scores in subjects with ADHD, the literature points to both reaction time and omission errors as being factors for this finding. Barkley [12] describes results of errors of omission on the Conners CPT as being indicative of inattention. It is this type of inattention that occurs during monotonous tasks (such as the Conners CPT) that we attempted to simulate in our monotonous segments. The results of the collisions appear to be a result of lack of attention.

The "object", which in the case of our simulator was a dog running across the road could have been a pedestrian, a child running after a ball or a bicyclist. Such results indicate the risk associated with impaired directed attention in drivers with ADHD can have serious consequences.

In contrast, results of the portions of the simulator designed to tease out impulsivity and hyperactivity did not result in abnormal findings when compared with Controls. The steering variability, rate of stopping and rate of passing under adverse conditions were all at similar levels to controls across the driving simulation. These results are consistent with the literature that purports that inattention is the dominant component of the clinical picture of adults with ADHD [24-26].

Our results should be considered in light of several methodological limitations. Since the majority of our subjects were Caucasians, our results may not generalize to other ethnic groups. Since our subjects were referred, our results may not generalize to community samples. Additionally, since our sample was relatively small, we may not have had sufficient power to detect small-sized effects on some outcomes. Also, our limited sample size precluded the examination of interactions between ADHD and age or psychiatric comorbidity on driving outcomes. Future research with larger samples should address these issues.

Despite the considerations, our results showed that ADHD subjects were selectively more likely than controls to collide with an obstacle, suddenly appearing from the periphery while driving in low stimulus environment. These results suggest that deficits in directed attention may underlie driving impairments in adults with ADHD and that such impairments in attention can result in serious accidents. Additional research is needed to evaluate whether intervention strategies can help correct ADHD-associated driving impairments and help prevent adverse occurrences. Considering the high prevalence of ADHD, such research would be of great clinical and public heath benefit.

## Competing interests

Dr. Joseph Biederman receives/d research support from the following sources: Shire, Eli Lilly, Pfizer, McNeil, Abbott, Bristol-Myers-Squibb, New River Pharmaceuticals, Cephalon, Janssen, Neurosearch, Stanley Medical Institute, Novartis, Lilly Foundation, Prechter Foundation, Astra-Zeneca, Forest Laboratories, Glaxo-SmithKline, NIMH, NICHD and NIDA. Dr. Joseph Biederman is a speaker for the following speaker's bureaus: Shire, Lilly, McNeil, and Cephalon, UCB Pharma, Inc, Novartis. Dr. Joseph Biederman is on the advisory board for the following pharmaceutical companies: Eli Lilly, Shire, McNeil, Janssen, Novartis, and Cephalon

Dr. Craig Surman has received research support from McNeil Pharmaceuticals, is on the speaker's bureau for Novartis Pharmaceuticals, and served in an advisory capacity to Shire Pharmaceuticals and Takeda Pharmaceuticals.

Dr. Thomas Spencer receives research support from the following sources: Shire Laboratories, Inc and Eli Lilly & Company, Glaxo-Smith Kline, Pfizer Pharmaceutical, McNeil Pharmaceutical, Novartis Pharmaceutical, and NIMH. Dr. Thomas Spencer is a speaker for the following speaker's bureaus: Glaxo-Smith Kline, Eli Lilly & Company, Novartis Pharmaceutical, Wyeth Ayerst, Shire Laboratories Inc, McNeil Pharmaceutical. Dr. Thomas Spencer is on the advisory board for the following pharmaceutical companies: Shire Laboratories, Inc and Eli Lilly & Company, Glaxo-Smith Kline, Pfizer Pharmaceutical, McNeil Pharmaceutical, and Novartis Pharmaceutical

Dr. Stephen V. Faraone receives research support from the following sources: Eli Lilly & Company, McNeil Consumer & Specialty Pharmaceuticals, Shire Pharmaceutical, the National Institute of Mental Health, the National Institute of Child Health and Development and the National Institute of Neurological Diseases and Stroke. Dr. Stephen V. Faraone is a speaker for the following speaker's bureaus: Eli Lilly & Company, McNeil Consumer & Specialty Pharmaceuticals, Novartis, Cephalon, Inc. and Shire Pharmaceuticals. Dr. Stephen V. Faraone has had an advisory or consulting relationship with the following pharmaceutical companies: McNeil Consumer & Specialty Pharmaceuticals, Shire Laboratories, Cephalon, Inc. and Eli Lilly & Company

## Authors' contributions

**JB**: Had full access to all the data in the study and take responsibility for the integrity of the data and the accuracy of the data analysis. I am also responsible for editing the final draft.

**RF**: Involved in the writing of the manuscript including reading the literature and integrating the literature with the findings and writing the discussion.

**MCM**: Provided the statistical analysis as well as integrating the information from the laboratory at M.I.T with the data at the laboratory at Massachusetts general Hospital.

**BR**: Worked closely with all authors on the design and analysis of the driving data.

**JFC**: Worked closely with all authors on the design and analysis of the driving data.

**CBS**: Involved in the writing of the manuscript including reading the literature and integrating the literature with the findings and writing the discussion.

**MA**: involved with recruitment of subjects, setting up the design of the study and editing the initial draft of the manuscript.

**MD**: involved with recruitment of subjects, setting up the design of the study and editing the initial draft of the manuscript.

**SS**: Generated the literature search, edited the paper for details and included all the references.

**TJS**: Involved in the writing of the manuscript including reading the literature and integrating the literature with the findings and writing the discussion.

**SVF**: Involved in the writing of the manuscript including reading the literature and integrating the literature with the findings and writing the discussion.
